# Manganese Porphyrin Treatment Improves Redox Status Caused by Acute Compressive Spinal Cord Trauma

**DOI:** 10.3390/antiox14050587

**Published:** 2025-05-14

**Authors:** Raquel Vieira Niella, Janaína Maria Xavier Corrêa, Claire Souza da Costa Marques, Álvaro José Chávez Silva, Luciano Cardoso Santos, Iago Santos de Oliveira, Gilson DeFreitas-Silva, Júlio Santos Rebouças, Juneo Freitas Silva, Mário Sérgio Lima de Lavor

**Affiliations:** 1 Department of Agricultural and Environmental Sciences, State University of Santa Cruz, Ilhéus 45662-900, BA, Brazil; jmxcorrea@uesc.br (J.M.X.C.); clairefaculdade@gmail.com (C.S.d.C.M.); ajcsilva@uesc.br (Á.J.C.S.); isoliveira.ppgca@uesc.br (I.S.d.O.); 2 Department of Biological Sciences, State University of Santa Cruz, Ilhéus 45662-900, BA, Brazil; lcsantos@uesc.br (L.C.S.); jfsilva@uesc.br (J.F.S.); 3 Department of Chemistry, Institute of Exact Sciences, Federal University of Minas Gerais, Belo Horizonte 30270901, MG, Brazil; gilsonufmg@ufmg.br; 4 Department of Chemistry, Institute of Exact and Natural Sciences, Federal University of Paraíba, João Pessoa 58051-970, PB, Brazil; jsreboucas@quimica.ufpb.br

**Keywords:** apoptosis, oxidative stress, endoplasmic reticulum stress, neuroprotection

## Abstract

There is increasing interest in identifying drugs that can prevent or delay neurological complications following spinal cord injury, thus expanding the therapeutic window for other potential neuroprotective agents. In this context, manganese porphyrins (MnPs) have shown high antioxidant and anti-inflammatory potential in various experimental disease models, including stroke, cancer, diabetes, ischemia, and radiotherapy. However, they have been little evaluated in spinal cord injuries. This study aimed to assess the therapeutic potential of the manganese porphyrins [MnTE-2-PyP]^5+^ (MnPI) and [MnT(5-Br-3-E-Py)P]^5+^ (MnPII) in acute compressive spinal cord trauma in rats. Twenty-four animals were used (six animals/group). Following general inhalation anesthesia, acute compressive spinal cord trauma was induced in all groups except for the negative control (SHAM). Treatment commenced 60 min post-trauma, with animals receiving treatment for seven days at 24 h intervals. While no improvement in motor capacity was observed, MnPs effectively blocked the increase in oxidative stress and endoplasmic reticulum (ER) stress mediators caused by trauma, maintaining the protein expression levels of Hifα, 8-OHdG and MDA, as well as the expression of the genes *Grp78*, *Chop*, *Ho1*, and *Perk*, similar to those of the control group. Moreover, there was an increase in protein expression of SOD1, Cat, and GPX1, along with a restoration of SOD and CAT enzymatic activity. Additionally, MnPs improved the expression of IL-6, neurotrophic markers, and apoptotic factors. In conclusion, treatment with MnPs attenuated the oxidative stress and ER stress caused by acute compressive spinal cord trauma and restored spinal expression of neurotrophic mediators.

## 1. Introduction

Spinal cord injuries, prevalent in both human and veterinary medicine, often result in severe consequences for affected individuals, including partial or complete loss of sensory, motor, and autonomic functions. Estimates suggest that around 2.5 million people worldwide are living with spinal cord injuries, with over 130,000 new cases reported annually [1,2].

Spinal cord injury, typically irreversible, results from a primary factor induced by mechanical forces, initiating a cascade of biochemical and cellular events that lead to secondary injury [3,4]. These secondary alterations manifest as various localized disturbances, including compromised circulation, ischemia, edema, and inflammation [5,6]. As a result, ischemic insults stemming from secondary events following mechanical spinal cord injury disrupt DNA synthesis, as well as protein and lipid synthesis and transport, leading to neuronal apoptosis and necrosis. Furthermore, these injuries impair myelination and synaptogenesis [7].

The central nervous system’s capacity for repair is severely limited following injury. Trauma triggers cell death, predominantly affecting neurons, oligodendrocytes, astrocytes, and microglia [8]. Prognosis remains poor, as current therapies yield limited neurological recovery, due to both primary injury and the progression of secondary damage, posing a significant treatment challenge. Consequently, this clinical condition continues to perplex researchers [9,10]. However, if treatment can successfully inhibit or mitigate secondary injury, the outlook for neurological recovery may improve considerably [8].

Research suggests that neurodegenerative and spinal disorders stem from an imbalance in cellular redox state, leading to systemic or localized inflammation caused by oxidative stress. Consequently, the primary goal in treating spinal cord injury patients is to inhibit or mitigate cellular changes that trigger secondary events, thereby promoting neurological recovery. This oxidative stress is directly linked to the cascade of biochemical and cellular events that exacerbate secondary damage following spinal cord injuries. Thus, antioxidant agents are targeted at neutralizing these oxidative processes, which are central to cellular damage. Their role focuses primarily on preventing neuronal apoptosis and necrosis, as well as preserving structural integrity, thereby fostering conditions that enable neurological recovery [11,12,13].

Water-soluble, cationic Mn porphyrins (MnPs) derived from 2-N-alkylpyridylporphyrins are potent mimics of the superoxide dismutase (SOD) enzymes and have shown significant therapeutic promise in managing oxidative stress in stroke, renal ischemia, and radioprotection models. Their effectiveness stems from potent antioxidant properties, minimal toxicity, and enhanced bioavailability due to their lipophilic nature [14,15]. To the best of our knowledge, the study on MnPs as redox-active therapeutics in spinal cord injuries is rather limited [16,17,18,19,20]. Of note, the efficacy of a Mn(III) N,N’-dialkylimidazolyporphyrin ([MnTDE-2-ImP^5+^]) in ameliorating spinal cord compression injury (SCC) in a mouse model was dependent on the MnP route of administration; rotarod performance was significantly improved with intrathecal administration of this MnP, whereas intravenous administration resulted in no effect [16].

Manganese porphyrins can regulate redox-dependent transcription factors like HIF1α and nuclear factor kappa NF-κB, while also suppressing inflammatory processes triggered by oxidative stress. Notably, even when MnPs are administered hours or weeks after the onset of oxidative stress, they can still provide significant protective effects [17].

Furthermore, [MnTE-2-PyP]^5+^ (MnPI) and [MnT(5-Br-3-E-Py)P]^5+^ (MnPII) have recently been shown to improve fetal–placental development of hypothyroid rats and protect against oxidative stress and ER stress caused by hypothyroidism at the maternal–fetal interface, with MnPI showing better effects than MnPII [21]. However, MnPII has not yet been described in the literature in other experimental models. Thus, this study aimed to assess the therapeutic potential of two MnPs formulations, MnPI and MnPII, in treating rats with acute compressive spinal cord injury.

## 2. Materials and Methods

### 2.1. Animals

Twenty-four adult male Wistar rats (*Rattus norvegicus*) (315 ± 45 g) were used, sourced from the Animal Facility of the State University of Santa Cruz (UESC). The rats were housed in plastic cages, with an average of four animals per cage, in a controlled environment maintaining a 12:12 light/dark cycle, temperature of 24 ± 1 °C, and humidity of 50 ± 5%. They were provided with daily water and ad libitum access to commercial rodent feed. All rats underwent a two-week acclimatization period prior to the experiment. All experimental procedures were approved by the Ethics Committee on Animal Use (CEUA) of the UESC (Protocol No. 038/20) and were performed according to the Animal Research: Reporting of In Vivo Experiments (ARRIVE) guidelines (https://arriveguidelines.org/ accessed on 10 November 2024) and the International Council for Laboratory Animal Science (ICLAS).

### 2.2. Synthesis and Characterization of Manganese Porphyrins (MnPs)

The two manganese porphyrins, [MnTE-2-PyP]^5+^ (MnPI) and [MnT(5-Br-3-E-Py)P]^5+^ (MnPII), both in aqueous solution, were synthesized and characterized as described in previous works [21,22,23,24,25,26].

### 2.3. Acute Compressive Spinal Cord Trauma Model

**Figure A1:**
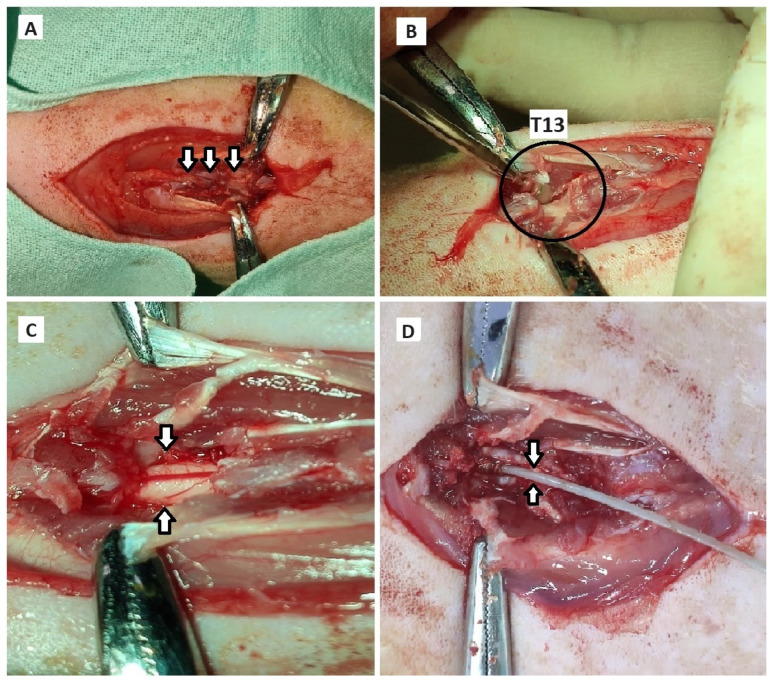
Photographic documentation of the experimental spinal cord injury surgical procedure in Wistar rats. (**A**) Retraction of epiaxial muscles and identification of spinous processes (arrow). (**B**) Dorsal lamina wear at T13 using a pneumatic drill (circle). (**C**) Visualization of the normal-appearing spinal cord following dorsal laminectomy (arrow). (**D**) Spinal cord injury induction via insertion of a Fogarty catheter into the spinal canal (arrow). Source: personal archive.

To induce acute compressive spinal cord injury, we employed the technique described by Khan and Griebel [27] (Figure A1). The animals were pre-medicated with morphine hydrochloride (morphine, 10 mg/mL, Cristália, Pharmaceutical Chemical Products Ltda, São Paulo, Brazil) at a dose of 5 mg/kg subcutaneously (SC) approximately 15 min prior to the procedure. They were then subjected to general inhalation anesthesia using sevoflurane (sevoflurane 1 mg/mL, Cristália Pharmaceutical Products Ltd.a, São Paulo, Brazil). The initial concentration for anesthetic induction was 4 V%, followed by 2.5 V% during maintenance to ensure the animals remained in an appropriate anesthetic-surgical plane.

All subjects were administered sodium cefalotin (ceflen^®^, Agila Pharmaceutical Products Ltd., Rio de Janeiro, Brazil) at 60 mg/kg/SC for prophylactic antibiotic treatment. Throughout surgery and anesthetic recovery, the animals were maintained at 38 °C on a heated platform, with core body temperature continuously monitored via rectal thermometry. Subjects breathed spontaneously through a facial mask delivering 100% oxygen.

Following extensive shaving of the animal’s back and antiseptic preparation, a 5 cm skin incision was made using the spinous processes of the tenth thoracic vertebra (T10) and the third lumbar vertebra (L3) as landmarks. The subcutaneous tissue was then dissected, and the epiaxial muscle insertions were incised and retracted laterally (Figure A1A). Using Kocher hemostatic forceps, the spinous process of the thirteenth thoracic vertebra (T13) was removed through ostectomy. Subsequently, the dorsal lamina of the process was worn down using a surgical drill (Figure A1B).

The spinal cord was visualized (Figure A1C), and, to induce acute compressive spinal cord injury, a Fogarty catheter No. 2 was inserted into the epidural space (Figure A1D) and guided caudally to the L1–L2 vertebral segment. The catheter balloon was inflated with 30 µL of 0.9% saline solution, and pressure was maintained on the spinal cord for five minutes. Subsequently, the balloon was deflated and the catheter removed. The muscle and subcutaneous tissue were approximated using a simple continuous suture pattern with 3-0 polyamide thread, and the skin was closed with the same thread in a simple interrupted pattern.

Following the surgical procedure, all animals were administered 0.9% saline solution subcutaneously at a maintenance rate of 15 mL/kg. For postoperative pain management, morphine was administered subcutaneously at a dose of 5 mg/kg every 8 h during the initial 24 h period. Throughout the entire 7-day evaluation period, bladder massage was performed every 8 h to ensure complete emptying of the urinary bladder. The animals were closely monitored for any signs of discharge, surgical site infection, or urinary tract infection.

### 2.4. Experimental Design

**Figure 1:**
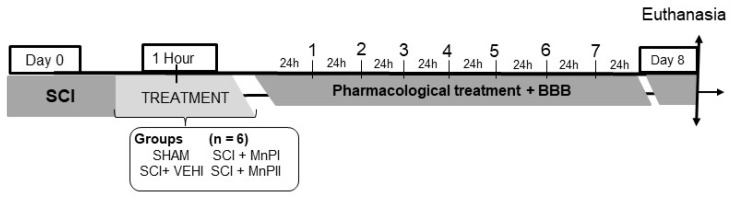
Representative diagram of the experimental schedule.

The animals were randomly assigned to four groups (n = 6 per group), each receiving the following treatments (Figure 1):

- Negative control (SHAM)—The subjects underwent dorsal laminectomy at T13 without spinal cord injury. One hour post-procedure, 0.9% NaCl solution (0.15 mL/100 g) was administered intraperitoneally.
- Positive control (SCI + VEHI)—The subjects underwent T13 dorsal laminectomy and spinal cord injury. One hour post-injury, 0.9% saline solution (0.15 mL/100 g) was administered intraperitoneally.
- Manganese Porphyrin I (SCI + MnPI)—The subjects underwent a dorsal laminectomy at the T13 vertebra, followed by spinal cord injury. One hour post-injury, MnPI—MnTE-2-PyP]^5+^—was administered intraperitoneally at a dose of 0.1 mg/kg/day. The dose was defined according to the Hambright [26], Batinic-Haberle [22], Rebouças [23], Rebouças [24], Pinto [25], and Cordeiro [21] studies.
- Manganese porphyrin II (SCI + MnPII)—The subjects underwent dorsal laminectomy at T13 and spinal cord injury. One hour after the injury, MnP II—[MnT(5-Br-3-E-Py)P]5+—(0.1 mg/Kg/day) was administered via IP every 24 h for seven days. The dose was defined according to the Cordeiro [21] study.

### 2.5. Motor Skills Assessment

Motor capacity evaluation was conducted 24 h prior to surgery to acclimate the animals, thereby minimizing stress and handling anxiety while facilitating post-operative assessments. The Basso, Beattie, and Bresnaham [28] (BBB scale) was employed, observing the animals’ locomotor patterns in an unobstructed, circular open field measuring one meter in diameter.

Over an 8-day period, the animals were assessed every 24 h. They were filmed for two minutes, and the footage was later analyzed by two trained evaluators who were unaware of the animals’ group assignments (blind study). The evaluators assigned scores ranging from 0 to 21, where 0 indicated a complete lack of motor function and 21 represented the highest possible score.

### 2.6. Necropsy and Material Collection

The rats were euthanized with a propofol overdose. The spinal cord was immediately collected after the death of each animal and divided into three fragments for subsequent analyses. The proximal fragment (10–20 mm proximal to the spinal cord lesion) was stored in a microtube containing TRIzol (Invitrogen, Life Technologies, Carlsbad, CA, USA) and immediately frozen in liquid nitrogen and stored at −80 °C for subsequent Reverse Transcription Quantitative Real-Time Polymerase Chain Reaction (RT-qPCR). The intermediate fragment (injured fragment) was fixed in 4% paraformaldehyde at 4 °C for 24 h and processed using the paraffin embedding technique. The tissues were dehydrated in solutions with increasing concentrations of alcohol (70% to 100%), followed by clearing in xylene and impregnation and inclusion in paraffin. Histological sections of four μm-thick tissues were obtained by microtomy on histological slides and stained with hematoxylin and eosin (H&E) for histological analyses. Gelatinized slides were used for immunohistochemistry (IHC). The distal fragment of the spinal cord was immediately frozen in liquid nitrogen and stored at −80 °C for subsequent evaluation of enzyme activities of SOD and catalase.

### 2.7. Immunohistochemistry (IHC)

Histological sections of the spinal cord were subjected to immunohistochemical analysis. The antibodies used were anti-8-OhdG (1:1000; sc-393871), anti-MDA (1:3000, sc-71136), anti-HIF1α (1:3000, sc-13515), anti-SOD1 (1:15000, sc-101523), anti-CAT (1:3000, sc-271803), anti-GPX1 (1:5000, sc-133160), anti-GRP78 (1:3000, sc-13539), anti-CHOP (1:3000, sc-71136), anti-IL-6 (1:3000, sc-365858), and anti-NeuN (1:8000, sc-365858), all from Santa Cruz Biotechnology, CA, USA.

The streptavidin biotin peroxidase technique was used (Streptavidin Peroxidase, Lab Vision Corp., Fremont, CA, USA), and antigen recovery was carried out by heating in a water bath at 98 °C, using a citric acid solution (0.54 M; pH 6.0). The slides were incubated overnight in a humid chamber with the primary antibody, and control sections, with the primary antibody replaced by phosphate-buffered saline (PBS), were included in all the reactions to check the specificity of the labeling. Incubation with the secondary antibody took place for 45 min, followed by incubation with streptavidin peroxidase for 30 min. The chromogen used was diaminobenzidine (DAB Substrate system, Lab Vision Corp., Fremont, CA, USA). Finally, the sections were counterstained with Harris hematoxylin.

The immunostaining area was quantified using WCIF ImageJ^®^ software version 1.41 (Media Cybernetics Manufacturing, Rockville, MD, USA) on random photomicrographs captured from five spinal cord grey matter using a Leica DM 2500 microscope equipped with a Leica DFC 295 digital camera (Leica Microsystems, Wetzlar, Germany). Image analysis involved color deconvolution and thresholding. Tissue data were archived, analyzed, and expressed as immunostaining area in pixels [29].

### 2.8. Real-Time Polymerase Chain Reaction (RT-qPCR)

One microgram of RNA was used for reverse transcription reactions using the commercial GoTaq^®^ qPCR and RT-qPCR Systems kit (A6010, PROMEGA). Target gene transcripts were quantified by qPCR using SYBR Green on the Applied Biosystems^®^ 7500 Real-Time PCR System. Each reaction contained 1.5 μL of cDNA, 100 nM of each primer, and 10 μL of GoTaq^®^ qPCR Master Mix (2×) in a final volume of 20 μL. A negative control was included using the DNA amplification mix with water substituting the cDNA sample. Primers for *Grp78*, *Chop*, *Hif1α*, *Perk*, *Nrf2*, *Ho1*, *Gdnf*, *Ngf*, *Gpx1*, *Sod1*, *Cat*, *Casp3,* and *Casp9* were designed based on Rattus norvegicus mRNA sequences (Table 1). Gene expression was calculated using the 2^−ΔΔCT^ method [30], and results for each group were quantitatively compared after normalization to Rattus norvegicus *Gadph* expression.

### 2.9. Evaluation of the Enzymatic Activity of SOD and Catalase

Spinal cord samples were homogenized with 50 nmoL of potassium phosphate buffer (TFK, pH = 7.0) and centrifuged at 13,400 rpm at 4 °C for 10 min to collect the supernatants. Protein concentration was evaluated by the Bradford method [31], while the enzymatic activities of SOD and catalase were evaluated according to Marklund and Marklund [32] and Aebi [33], respectively.

### 2.10. Statistical Analysis

Statistical analysis was conducted using GraphPad Prism Software^®^ version 8.0.2. A completely randomized design was employed, with results expressed as mean ± standard error of the mean (SEM). Animal motor capacity data (BBB scale) were analyzed using two-way analysis of variance (ANOVA) followed by Dunnett’s post-hoc test, while Student’s *t*-test was utilized for comparisons between two groups. IHC, RT-qPCR, and enzyme activity data were subjected to one-way ANOVA followed by the Student–Newman–Keuls (SNK) post-hoc test. The significance level was set at 5% (*p* < 0.05).

## 3. Results

### 3.1. Motor Capacity Assessment

Prior to surgery, all animals exhibited normal neurological parameters, achieving the maximum score of 21 points on the BBB scale, confirming the absence of pre-existing deficits. Acclimating the animals before the surgical procedure proved crucial in minimizing stress during assessments.

Following the surgical procedure and throughout all evaluations, the SHAM (laminectomy) animals demonstrated no neurological deficits. During the BBB assessment, they displayed normal gait, full weight-bearing on all four limbs, raised tail, and trunk stability—characteristics that align with the maximum score of 21.

**Figure 2:**
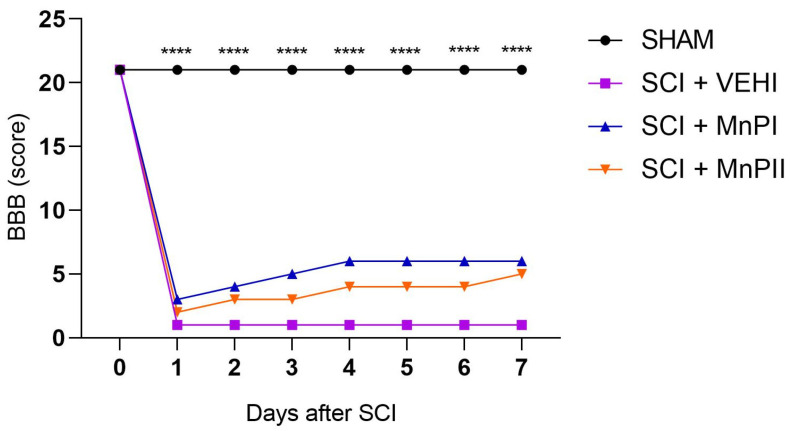
Motor function evaluation. Median daily scores of Wistar rats undergoing laminectomy (SHAM), acute compressive spinal cord injury (SCI + VEHI), and acute compressive spinal cord injury treated with MnPI and MnPII F (3, 21) = 47.30. (**** *p* < 0.0001).

The BBB assessment revealed a statistically significant difference between the SHAM group and the other groups (Figure 2). In the first evaluation, performed 24 h after the initial surgical procedure, the SHAM animals achieved maximum scores, while the other groups exhibited severe neurological deficits. These deficits included paraplegia and scores indicating absent or minimal movement in one or two joints of the examined pelvic limb. No statistical difference was observed among the groups that experienced spinal cord injury (SCI + VEHI) and those that received treatment (SCI + MnPI and SCI + MnPII) (Figure 2).

Seven days post-acute compressive spinal cord injury, the groups that underwent spinal trauma and were treated with Porphyrins exhibited scores indicative of early-stage recovery in the BBB test (score 5), although this was not statistically significant. Their pelvic limbs dragged along the ground while walking, displaying either no joint movement or limited to extensive joint mobility, whereas the untreated spinal cord injury group remained with severe neurological deficits (score 0).

### 3.2. Anatomopathological Evaluation of the Spinal Cord

Upon macroscopic examination, the SHAM group animals exhibited no discernible alterations in their spinal cords. The spinal cords in this group displayed a characteristic whitish hue and lacked meningeal adhesions at the laminectomy site. Conversely, the SCI + VEHI, SCI + MnPI, and SCI + MnPII groups presented with localized areas of adhesion between the dura mater and vertebrae, accompanied by spinal cord hyperemia. These conditions significantly complicated the dissection process.

**Figure 3:**
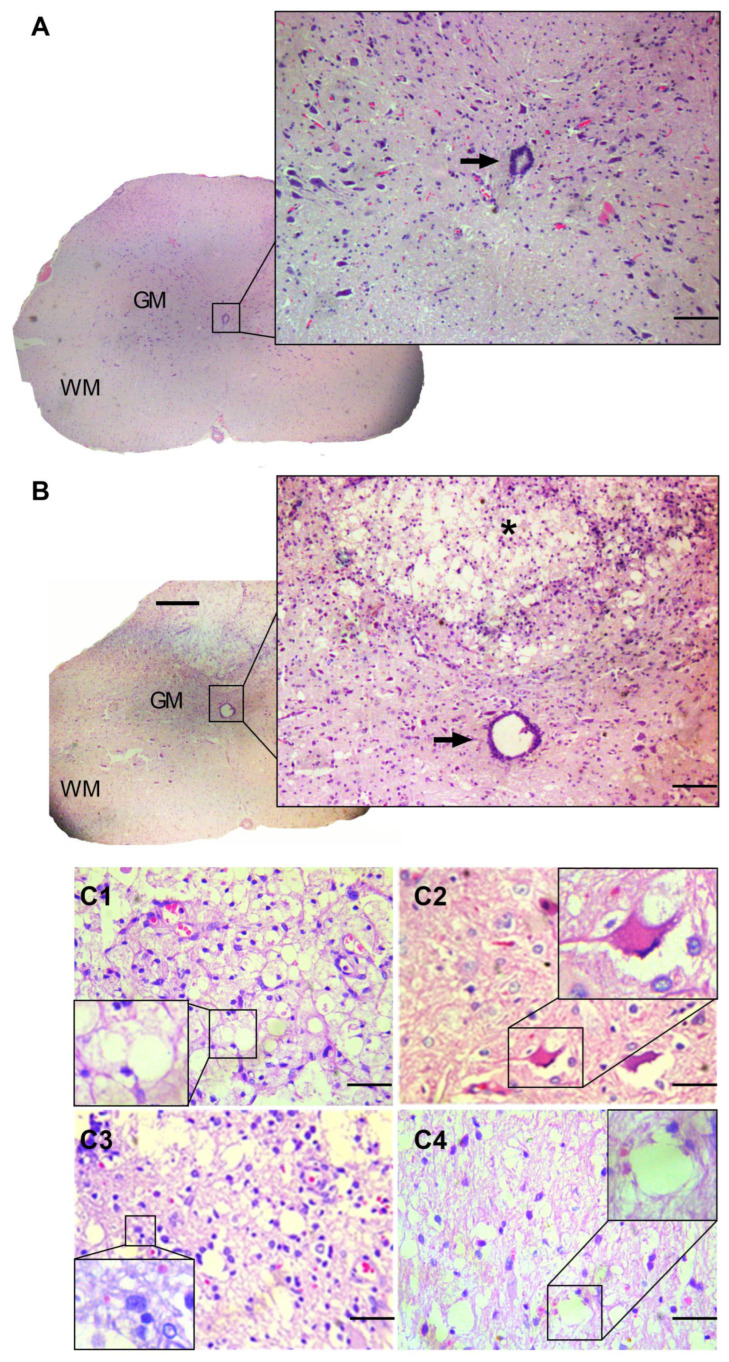
Photomicrograph of a Wistar rat spinal cord cross-section stained with hematoxylin and eosin (H&E). (**A**) Morphologically normal spinal cord from the SHAM group, showing distinct white matter (WM) and gray matter (GM). The arrow indicates the ependymal canal. (**B**) Altered spinal cord morphology in the SCI + VEHI group, exhibiting pronounced multifocal areas of malacia in the white matter and portions of the gray matter (asterisk) dorsal to the ependymal canal. (**C1**) Axonal degeneration in a segment adjacent to the injury epicenter (highlight); (**C2**) neuronal necrosis and chromatolysis (highlight); (**C3**) gliosis (highlight); (**C4**) axonal degeneration within an area of malacia (highlight). 40× magnification.

Microscopic examination revealed preserved architecture in the SHAM group (Figure 3A). The dura mater and leptomeninges were observed encasing the rounded spinal cord, which was divided into an “H”-shaped gray matter core surrounded by white matter. The gray matter primarily consisted of neuronal cell bodies and lightly myelinated axons, while the white matter contained myelinated axons. Centrally positioned was the central canal, lined with ependymal cells. The trauma-subjected groups (SCI + VEHI, SCI + MnPI, and SCI + MnPII) exhibited comparable histological alterations (Figure 3B). Severe diffuse malacia, marked by cellular depletion and cavitation areas, was evident in the epicenter region.

As the lesion spread to neighboring areas, the dorsal funiculus exhibited mild to moderate and focal to focally extensive softening of tissue, accompanied by cellular debris, cavitation, and infiltration of activated microglia or gitter cells. Additionally, axonal degeneration and myelin swelling were observed (Figure 3C1), along with neuronal chromatolysis (Figure 3C2), gliosis (Figure 3C3), and the formation of digestion chambers (Figure 3C4).

### 3.3. MnP Treatment Mitigates Oxidative Damage by Inhibiting the Spinal Elevation of 8-OHdG, MDA, and HIF1α in Rats Following Spinal Cord Trauma

**Figure 4:**
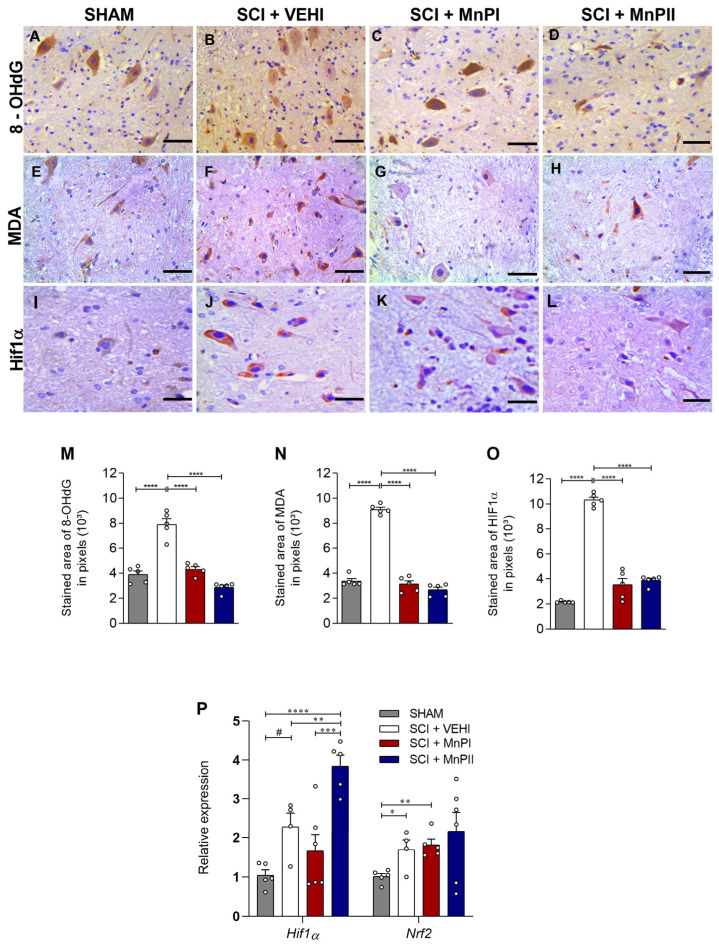
Effects of MnPs treatment on the expression of cellular oxidative damage markers in the spinal cord of rats subjected to trauma. (**A**–**L**) Photomicrographs of the immunolabeling of 8-OHdG (**A**–**D**), MDA (**E**–**H**), and HIF1α (**I**–**L**) in the spinal cord of rats from the SHAM (**A**,**E**,**I**), SCI + VEHI (**B**,**F**,**J**), SCI + MnPI (**C**,**G**,**K**), and SCI + MnPII (**D**,**H**,**L**) groups. 40× magnification. (Hematoxylin; bar = 50 µm). (**M**–**O**) Area of immunolabeling of 8-OHdG (**M**) F (3, 16) = 50.82, MDA (**N**) F (3, 16) = 201.3 and HIF1α (**O**) F (3, 16) = 155.6; (**P**) relative gene expression (fold change) of *Hif1α* F (3, 16) = 13.58 and *Nrf2* F (3, 16) = 2.38 in the spinal cord of rats submitted to trauma, treated or not with MnPI and MnPII (mean ± SEM; * *p* < 0.05; ** *p <* 0.01; *** *p* < 0.001; **** *p* < 0.0001; # *p* < 0.05 test *t*).

We initially aimed to assess hypoxia and oxidative damage in the spinal cord of the animals. This was achieved through immunohistochemical analysis of several markers: 8-hydroxyl-2′-deoxyguanosine (8-OHdG), an indicator of oxidative DNA damage [33]; malondialdehyde (MDA), a product of lipid peroxidation; HIF1α, a hypoxia marker [34]; and Nrf2, a transcription factor involved in the expression of antioxidant enzymes under hypoxic conditions [35]. Notably, the SCI + VEHI animals (Figure 4B) exhibited a characteristic pattern of oxidative damage, with significantly higher levels of 8-OHdG, MDA, and HIF1α compared to the SHAM group (Figure 4M–O). Treatment with both MnPs I and II effectively prevented the elevated spinal expression of these markers (*p <* 0.001), bringing them down to levels comparable to those observed in the SHAM group. With respect to *Hif1α* gene expression, no significant difference was observed between SHAM and SCI + VEHI groups (Figure 4P), while the SCI + MnPII group showed a significantly higher expression compared to the other groups.

In relation to Nrf2, the analysis revealed that spinal cord injury (SCI + VEHI) significantly elevated transcript expression compared to the control group (*p* = 0.02). However, no significant differences were observed in the treated animals (Figure 4P).

### 3.4. MnP Treatment Enhances Antioxidant Enzyme Protein Expression and Activity in Rats Following Spinal Cord Trauma

Given the increased expression of *HIF1α* and *Nrf2* in trauma-subjected animals and the reduction in HIF1α expression following MnPI and MnPII treatments, we assessed the expression and/or enzymatic activity of GPx1, catalase, and SOD1—the primary antioxidant enzymes responsible for managing oxidative stress under hypoxic conditions [36].

**Figure 5:**
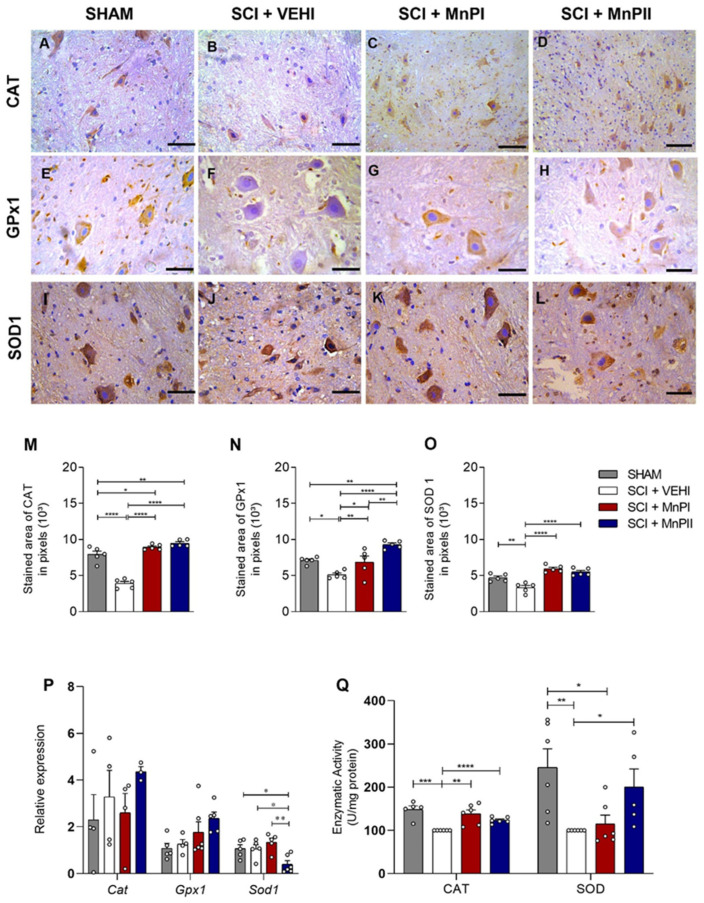
Effects of MnPs treatment on the expression of antioxidant mediators in the spinal cord of rats subjected to trauma. (**A**–**L**) Photomicrographs of CAT (**A**–**D**), GPX1 (**E**–**H**), and SOD 1 (**I**–**L**) immunolabeling in the spinal cord of rats from the SHAM (**A**,**E**,**I**), SCI + VEHI (**B**,**F**,**J**), SCI + MnPI (**C**,**G**,**K**), and SCI + MnPII (**D**,**H**,**L**) groups. 40× magnification. (Hematoxylin; Bar = 50 µm). (**M**–**O**) Area of immunolabeling of CAT (**M**) F (3, 16) = 67.91, GPX1 (**N**) F (3, 16) = 13.68 and SOD 1 (**O**) F (3, 16) = 21.30; (**P**) relative gene expression (fold change) of *Cat* F (3, 11) = 1.64, *Gpx1* F (3, 16) = 2.95, and *Sod1* F (3, 17) = 6.16; (**Q**) Enzymatic Activitys in the spinal cord of rats subjected to trauma, treated or not with MnPI and MnPII (mean ± SEM; * *p* < 0.05; ** *p* < 0.01; *** *p* < 0.001; **** *p* < 0.0001).

Immunohistochemical analysis of these markers revealed that the trauma-subjected animals (SCI + VEHI) exhibited a significant decrease in immunoreactivity compared to the control group (Figure 5B,F,J). Treatments with MnPs I and II enhanced the immunolabeling of Cat, GPx1, and SOD1 in the spinal cord (Figure 5M–O), effectively restoring the labeling pattern. On the other hand, the gene expression levels of *Cat* and *GPx1* (Figure 5P) showed no significant differences among groups, whereas MnP II significantly decreased the medullary expression of *Sod1* transcripts.

In terms of catalase and SOD enzymatic activity in the spinal cord, a significant increase in enzyme consumption was observed in SCI + VEHI compared to the control, as evidenced by reduced activity. MnPs treatments successfully restored the enzymatic activity altered by spinal cord trauma to control levels for catalase and SOD (Figure 5Q).

### 3.5. MnP Treatment Inhibits the Elevation of Unfolded Protein Response (UPR) Mediators in Rats Following Spinal Cord Trauma

Acute compressive spinal cord injury induces ER stress in rat spinal cords [33]. We aimed to investigate whether treatment with MnPs I or II could prevent this process. To achieve this, we analyzed *Grp78*, *Chop*, *Perk*, and *Ho1*, which are key mediators of the UPR pathway and indicators of ER stress.

**Figure 6:**
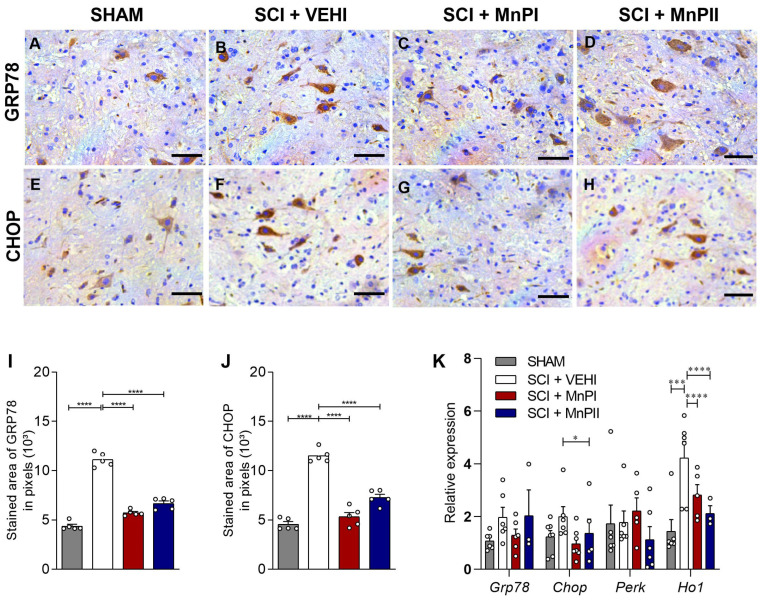
Effects of MnPs treatment on the expression of UPR mediators and endoplasmic reticulum stress in the spinal cord of rats subjected to trauma. (**A**–**H**) Photomicrographs of GRP78 (**A**–**D**), CHOP (E-H) immunolabeling in the spinal cord of rats from the SHAM (**A**,**E**), SCI + VEHI (**B**,**F**), SCI + MnPI (**C**,**G**), and SCI + MnPII (**D**,**H**) groups. 40× magnification. (Hematoxylin; Bar = 50 µm). (**I**,**J**) Area of GRP78 immunolabeling (**I**) F (3, 16) = 155.9, CHOP (**J**) F (3, 16) = 83.20; (**K**) relative gene expression (fold change) of *Grp78* F (4, 25) = 2.41, *Chop* F (4, 16) = 1.93, *Perk* F (3, 16) = 1.83, and *Ho1* F (3, 16) = 28.64 in the spinal cord of rats submitted to trauma, treated or not with MnPI and MnPII (mean ± SEM; * *p* < 0.05; *** *p* < 0.001; **** *p* < 0.0001).

The GRP78 and CHOP proteins exhibited enhanced medullary immunolabeling in the SCI + VEHI group (Figure 6B). However, treatments with MnPs I and II reversed the trauma-induced increase in expression (Figure 6I,J), bringing it back to levels comparable to the control group. As for *Chop* gene expression, both MnPs I and II treatments mitigated the trauma-induced increase, with a significant difference observed between MnPII and SCI + VEHI (Figure 6K).

Spinal cord injury enhanced *Ho1* gene expression (Figure 6K) compared to the SHAM group. Both MnP’s treatments significantly decreased the expression of *Ho1* (Figure 6K). No significant differences in the expression of *Grp78* and *Perk* were observed between the groups.

### 3.6. MnP Treatment Changes IL-6, NeuN, Gdnf, Casp 3, and Casp 9 Expression in Rats Following Spinal Cord Trauma

**Figure 7:**
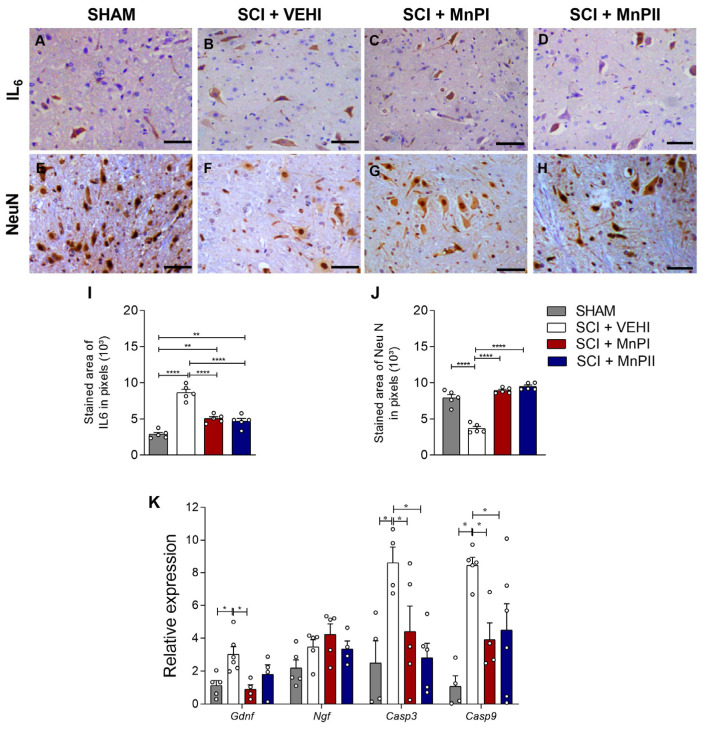
Effects of MnPs treatment on the expression of immunological and apoptotic mediators in the spinal cord of rats subjected to trauma. (**A**–**H**) Photomicrographs of IL-6 (**A**–**D**), NeuN (**E**–**H**) immunolabeling in the spinal cord of rats from the SHAM (**A**,**E**), SCI + VEHI (**B**,**F**), SCI + MnPI (**C**,**G**), and SCI + MnPII (**D**,**H**) groups. 40× magnification. (Hematoxylin; Bar = 50 µm). (**I**,**J**) Area of immunolabeling of IL-6 (**I**) F (3, 16) = 46.31, NeuN (**J**) F (3, 16) = 73.58; relative gene expression (fold change) of *Gdnf* F (3, 14) = 4.67, *Ngf* F (3, 15) = 2.71, *Casp3* F (3, 14) = 4.77, and *Casp9* (**K**) F (3, 15) = 6.35 in the spinal cord of rats subjected to trauma, treated or not with MnPI and MnPII (mean ± SEM; * *p* < 0.05; ** *p <* 0.01; **** *p* < 0.0001).

Concerning IL-6, spinal cord injury led to a significant increase in immunoreactivity in the SCI + VEHI group (Figure 7B) compared to the control. Conversely, MnPs treatments successfully diminished and restored the staining pattern (Figure 7I). In terms of neuronal nuclear protein (NeuN) immunostaining, a reduced expression was noted in the SCI + VEHI group compared to the SHAM group (Figure 7F). Conversely, treatments with MnPs led to a significant increase in immunostaining (Figure 7J).

Concerning *Casp3* and *Casp9* expression, both porphyrins significantly decreased the elevated spinal cord expression induced by trauma (Figure 7K). Collectively, these findings demonstrate that spinal cord trauma increases the expression of pro-inflammatory and apoptotic markers, and that MnP treatments effectively reduce this response. Concerning the neurotrophic gene *Gdnf*, a significant increase was observed in the SCI + VEHI group. However, MnPI treatment led to reestablished levels equaling the SHAM group in expression (Figure 7K). There were no significant differences in the expression of *Ngf* between the groups (Figure 7K).

## 4. Discussion

This study demonstrated that the administration of manganese metalloporphyrins [MnTE-2-PyP]^5+^ (MnPI) or [MnT(5-Br-3-E-Py)P]^5+^ (MnPII) did not enhance motor function in rats with acute compressive spinal cord injury after seven days of treatment. Nevertheless, they protected the nervous tissue surrounding the injury epicenter against hypoxia, oxidative stress, ER stress, and apoptosis development. This research contributes to the evaluation of MnP’s therapeutic potential in acute compressive spinal cord injury associated with spinal stress using an experimental model; to the best of our knowledge, there has been only one prior evaluation of the effect of a related MnP ([MnTDE-2-ImP^5+^]) on ameliorating spinal cord compression injury in a mouse model assessed by rotarod performance [16].

The BBB scale motor capacity assessment test was selected as a widely recognized and commonly used tool for evaluating outcomes, progression, and recovery of experimental spinal cord injuries in rats [37,38,39]. This scale is a sensitive, valid, and reliable measure of rat locomotor function [40]. Substantial evidence indicates that the degree of locomotor function deficit, spinal cord edema level, and apoptotic cell death correlate with the severity of secondary neural injury induced by trauma [41,42]. Groups subjected to acute compressive spinal cord trauma showed significantly decreased scores, consistent with findings from studies using the same experimental model [43,44]. However, under the tested conditions, MnPs did not improve the animals’ locomotor function, likely due to the severity of the injury and short treatment time.

The initial assessment, performed 24 h post-surgery, yielded a score of 21 in the SHAM group. This result confirmed that the animals sustained no injuries from the surgical procedure used to access the spinal cord via dorsal laminectomy. It further indicates that the technique was executed with precision and care, aligning with findings reported in previous studies [45,46].

Oxidative stress plays a significant role in the mechanisms involved in the pathophysiological processes of spinal cord injury. Our study evaluated the markers 8-OHdG, MDA, and HIF1α, which are biomarkers of oxidative DNA damage, lipoperoxidation, and cellular hypoxia, respectively [47,48]. We observed that spinal cord injury led to dysregulation of these markers, resulting in elevated levels of 8-OHdG, MDA, and HIF1α. These findings corroborate other studies that have examined the expression of these markers in the same experimental model [34,49,50,51].

Studies on therapies that enhance antioxidant defenses or mitigate pro-oxidant processes have demonstrated prevention and improvement of neurological alterations, as well as neuroprotective effects [51]. In a positive way, MnPs successfully reduced the levels of the markers 8-OHdG, MDA, and HIF1α following spinal cord injury. A previous investigation using MnPI also showed decreased protein expression of 8-OHdG and HIF1α in orthotopic 4T1 breast carcinomas in 20 Balb/c mice, indicating a reduction in hypoxia and oxidative stress in this model [52].

In a radiation-induced lung injury model using MnPI in female rats, Gauter-Fleckenstein [53] also demonstrated reduced immunostaining of HIF1α and 8-OHdG. Collectively, these findings confirm that MnPsI and II offer protective effects against hypoxia and oxidative stress in the spinal cord resulting from acute compressive spinal cord trauma. Notably, MnPII exhibits cytoprotective effects comparable to MnPI in combating oxidative stress, which is consistent with our previous results in a rat maternal hypothyroidism model [21].

The observed reduction in hypoxia and oxidative damage may result from the direct action of these MnPs, which demonstrate SOD-mimetic activity and potent catalytic action against reactive oxygen and nitrogen species, especially superoxide and peroxynitrite, causing signaling and possibly a reduction in the expression of these factors [54]. While treatments with MnPsI and II did not reverse the gene expression of *Nrf2*, a transcription factor involved in antioxidant enzyme expression [55], both porphyrins enhanced the protein expression of CAT, SOD1, and GPx1/2 in injured rats. These enzymes play crucial roles in regulating reactive oxygen species in biological systems [56,57].

Our findings align with recent studies on MnPI, which revealed increased *Nrf2* expression in an in vitro model using irradiated mouse and human prostate fibroblasts [58] and under hyperglycemic conditions [59]. It is well-established that the activation of the *Nrf2* pathway by porphyrin-based SOD mimetics plays a crucial role in regulating antioxidant enzymes such as SOD, CAT, GPX, and MnSOD [60]. It is worth noting that our study only analyzed the medullary gene expression of *Nrf2*; immunostaining, as performed for antioxidant enzymes, would have provided a more comprehensive analysis of this factor’s expression. Moreover, the studies by Shrishrimal [61] and Chatterjee [62] were conducted in vitro, which may not fully capture the complexity of in vivo studies like ours.

Conversely, our findings on immunostaining and gene expression of antioxidant enzymes align with a study demonstrating that pretreatment with [MnTM-4-PyP]^5+^ in an in vitro model of oxidative stress in rat cortical neurons exposed to hydrogen peroxide (H_2_O_2_) significantly elevated protein levels of SOD1, SOD2, and CAT, while simultaneously reducing gene expression of *Sod1* and *Cat* [63]. The discrepancy between protein and mRNA levels may be attributed to the short half-life of mRNA or negative feedback on transcription [64,65,66]. Additionally, a previous study conducted by Zhao [67] showed that both in vitro and in vivo treatment with another MnP, [MnTnBuOE-2-PyP]^5+^, enhanced gene and/or protein expression of Cat and SOD in bone marrow cells of C57BL/6 mice. Our findings demonstrate that treatments with MnPsI and II offer protection against oxidative stress following acute compressive spinal cord injury in rats. These treatments reduce the expression of 8-OHdG, MDA, and HIF1α while increasing the protein expression of catalase, SOD1, and GPx1.

Although endoplasmic reticulum stress has not yet been studied in models of acute compressive spinal cord injury with MnPs, its potential protective effect was previously demonstrated in a study examining the maternal–fetal interface of hypothyroid rats [21]. Treatments with MnPsI and II mitigated the trauma-induced elevation of GRP78 and CHOP protein expression in the spinal cord, and treatment with MnPsII reduced the gene expression of Chop and Ho1. These findings are consistent with in vitro ER stress studies that demonstrated reduced expression of *Grp78* and/or *Chop* following [MnTM-4-PyP]^5+^ treatment in primary rat cortical neurons exposed to H_2_O_2_ [57] and after [MnTM-4-PyP]^5+^ administration in lung adenocarcinoma cells (A549) [68].

*Grp78* and *Chop* are established markers of endoplasmic reticulum stress activation [59,60], while *Ho1* is recognized for its crucial role in regulating oxidative stress [69,70]. Several studies have demonstrated elevated expression of these mediators following spinal cord injury [71,72,73]. This research underscores the potential of MnPsI and II as protective agents against ER stress in the spinal cord of rats following trauma.

Moreover, oxidative stress can aggravate or trigger various inflammatory and cellular apoptotic pathways. Consequently, inhibiting oxidative pathways may significantly mitigate the severity of spinal cord injury. IL-6 expression was elevated in the injury group, which is consistent with its role as a pro-inflammatory cytokine known to be upregulated in the vicinity of the damaged area and remain elevated [68]. Notably, treatments with MnPs effectively reduced the heightened immunostaining of spinal IL-6 in rats following injury.

High concentrations of NeuN expression indicate neuronal viability, serving as a crucial marker for assessing neuronal integrity and the positive effects on cell survival when subjected to experimental treatments [74,75]. Research has demonstrated that NeuN immunoreactivity decreases in pathological conditions that compromise neuronal viability, such as trauma, hypoxia, and cerebral ischemia [76], which aligns with our findings. Moreover, the administration of MnPsI and II enhanced NeuN expression in the post-traumatic spinal cord, indicating a potential neuroprotective effect. Although we did not evaluate the effect of these porphyrins on neuronal loss after spinal cord injury in this study, and considering that NeuN is a typical marker of mature neurons [76], it is possible that MnPs play a protective role in post-injury neuronal cell loss or that they somehow delay this damage. However, this hypothesis needs to be evaluated with this aim in future studies.

Caspases play a crucial role in cell survival and death [50]. Our study demonstrated that the injury model induced apoptosis, significantly increasing the expression of *caspases 3* and *9*. Notably, MnPs significantly reduced the gene expression of these proteins. While these results are not conclusive, they suggest that MnPs interfere with the apoptosis cascade and exhibit anti-apoptotic properties through mechanisms related to these factors.

Neurotrophins (*Ngf*, *Gdnf*, *Bdnf,* and *Nt-3*) are widely recognized for their crucial role in maintaining the balance between neuronal survival and apoptosis through their basal levels [77,78]. Spinal cord injury disrupts this equilibrium, leading to increased pro-neurotrophin expression, which accelerates apoptosis, reduces synaptic plasticity, and intensifies the overall inflammatory response [79,80,81,82]. Our study revealed elevated gene levels of *Gdnf* in the SCI + VEHI group, while MnPI treatment may have regulated this overexpression, bringing Gdnf levels back to normal (as observed in the SHAM group). This suggests that MnPI may reduce cellular stress and the need for such an intense compensatory response by the organism. MnPs treatments evidently play a significant role in the post-spinal cord injury apoptosis pathway, potentially contributing to the reduced spinal expression of IL-6 and *Gdnf*.

## 5. Conclusions

This study demonstrated that the manganese porphyrins MnPI, [MnTE-2-PyP]^5+^, and MnPII, [MnT(5-Br-3-E-Py)P]^5+^, did not improve locomotor deficits in acute compressive spinal cord trauma in rats after seven days of treatment, but they attenuated hypoxia, apoptotic mediators, inflammation, and redox dysfunction. These findings suggest that manganese porphyrins could serve as potential therapeutic alternatives for treating disorders arising from acute compressive spinal cord injury.

## Figures and Tables

**Table 1:** List of genes and nucleotide sequence of primers for RT-qPCR.

**Gene**	**Sequence (5->3)**	**Access No.**
*Grp78*	Forward: TGAAGGGGAGCGTCTGATTG Reverse: TCATTCCAAGTGCGTCCGAT	NM_013083.2
*Chop*	Forward: TGGCACAGCTTGCTGAAGAG Reverse: TCAGGCGCTCGATTTCCT	NM_001109986.1
*Perk*	Forward: GGCTGGTGAGGGATGGTAAA Reverse: TTGGCTGTGTAACTTGTGTCATC	NM_031599.2
*Ho1*	Forward: CAGCATACGTAAAGCGTCTCCA Reverse:CATGGCCTTCTGCGCAATCTTCTT	NM_012580.2
*Hifα*	Forward: AGCAATTCTCCAAGCCCTCC Reverse: TTCATCAGTGGTGGCAGTTG	NM_024359.1
*Nrf2*	Forward: CCCATTGAGGGCTGTGATCT Reverse: GCCTTCAGTGTGCTTCTGGTT	NM_031789.2
*Catalase*	Forward: CTGACTGACGCGATTGCCTA Reverse: GTGGTCAGGACATCGGGTTT	NM_012520.2
*Gpx1*	Forward: GCGCTACAGCGGATTTTTGA Reverse: GAAGGCATACACGGTGGACT	NM_030826.4
*Sod1*	Forward: GAAAGGACGGTGTGGCCAAT Reverse: CTCGTGGACCACCATAGTACGT	NM_017050.1
*Gdnf*	Forward: CAAGGTAGGCCAGGCATGTT Reverse: CACACCGTTTAGCGGAAT	NM_001401780.1
*Ngf*	Forward: CCTGGAGCCGAAGGGGA Reverse: CACTGAGGTGGAGCTTGGGTC	NM_001277055.1
*Caps3*	Forward: GAGCTTGGAACGCGAAGAAA Reverse: AGTCCATCGACTTGCTTCCA	NM_012922.2
*Casp9*	Forward: TCCCCACTGATCAAGTCTCCT Reverse: CCAGGCTCACTTAGCAAGGAA	NM_031632.2
*Gapdh*	Forward: GCGCTACAGCGGATTTTTGA Reverse: GAAGGCATACACGGTGGACT	NM_031797.2

## Data Availability

The raw data supporting the conclusions of this article will be made available by the authors upon request.
